# Mnemonic convergence in the human hippocampus

**DOI:** 10.1038/ncomms11991

**Published:** 2016-06-21

**Authors:** Alexander R. Backus, Sander E. Bosch, Matthias Ekman, Alejandro Vicente Grabovetsky, Christian F. Doeller

**Affiliations:** 1 Radboud University, Donders Institute for Brain, Cognition and Behaviour, Nijmegen 6525 EN, The Netherlands

## Abstract

The ability to form associations between a multitude of events is the hallmark of episodic memory. Computational models have espoused the importance of the hippocampus as convergence zone, binding different aspects of an episode into a coherent representation, by integrating information from multiple brain regions. However, evidence for this long-held hypothesis is limited, since previous work has largely focused on representational and network properties of the hippocampus in isolation. Here we identify the hippocampus as mnemonic convergence zone, using a combination of multivariate pattern and graph-theoretical network analyses of functional magnetic resonance imaging data from humans performing an associative memory task. We observe overlap of conjunctive coding and hub-like network attributes in the hippocampus. These results provide evidence for mnemonic convergence in the hippocampus, underlying the integration of distributed information into episodic memory representations.

## Abstract (web summary)

The ability to form associations between events is the hallmark of episodic memory and is thought to involve the hippocampus. Here the authors use a combination of multivariate pattern and graph theoretical network analyses of functional imaging data in humans, and show conjunctive coding and hub-like network attributes in the hippocampus.

## Graphical abstract

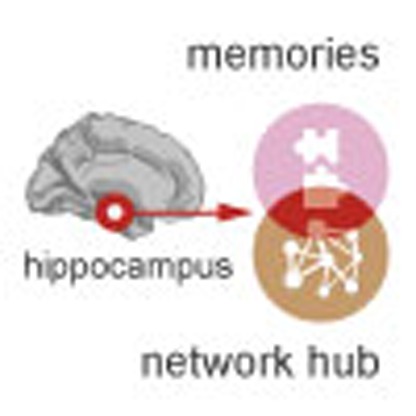

Episodic memories entail a rich set of different features, such as the place where an event occurred (for example, the local bakery), the people encountered (for example, a teacher from our children's school), the content of a conversation (for example, the upcoming Christmas party at school) and when it took place (for example, Wednesday afternoon). An important aspect of memory formation is the convergence of such separate elements onto a conjunctive representation^1^,^2^. This convergence of information is crucial not only for simple associations between stimulus features, but just as much for the binding of relationships between places, people, objects and events into complex episodic memories. But how does the brain implement mnemonic convergence? Computational models of memory have hypothesized for a long time that specialized modules, so-called convergence zones, exist in the brain^3^,^4^,^5^,^6^,^7^. These zones are characterized by two key properties: conjunctive coding and a high degree of interconnectivity with other brain regions. Although the existence of convergence zones is widely acknowledged, there is as of yet limited evidence for their neural underpinnings.

A prime candidate for mnemonic convergence is the hippocampus, a brain region that is thought to index the cortical elements of an episodic memory representation^7^,^8^,^9^ by means of conjunctive coding^3^. In line with this idea, several theories have posited a key role for the hippocampus in binding item and context information and binding of discontiguous elements^10^,^11^. Experimental evidence from studies using electrophysiological recordings^12^,^13^ and functional magnetic resonance imaging (fMRI) in humans^14^,^15^,^16^,^17^,^18^,^19^ support conjunctive representations in the hippocampus using a wide array of experimental tasks. However, many of these studies have pursued a region-of-interest approach, thus neglecting the network perspective. In parallel, the convergent connectivity profile of the hippocampus has been traditionally examined using neuronal tracer techniques in animals^20^,^21^ and neuroimaging connectivity methods in humans^22^,^23^,^24^. Although some studies investigate network properties during cognitive tasks^25^,^26^,^27^,^28^,^29^, many connectivity studies focus on the entire (often rodent or monkey) brain at rest, ignoring the relationship between brain connectivity and task-relevant, regionally specific representations. Thus, surprisingly, the two key properties that define a convergence zone, namely conjunctive representations (hereafter referred to as conjunctiveness) and interconnectivity with other brain regions (hereafter referred to as hubness), have hitherto been only studied in isolation in the human hippocampus.

Here we investigate whether the hippocampus is a convergence zone and test the prediction that the hippocampus plays a special role in associative binding. We use a simple associative learning paradigm and fMRI techniques, in combination with two analysis approaches to simultaneously gauge the two key properties of a convergence zone: we employ representational similarity analysis (RSA)^30^ to assess neural representation of conjunctiveness in regional multivoxel patterns and adopt a graph-theoretical network approach^31^ to quantify hubness from the functional connectivity data during memory retrieval. Subsequently, we assess the overlap of these two neural metrics as a marker of mnemonic convergence. Importantly, we employ whole-brain analyses to investigate a region-specific question: is the hippocampus a convergence zone, characterized by a combination of both conjunctiveness and hubness?

## Results

**Figure 1:**
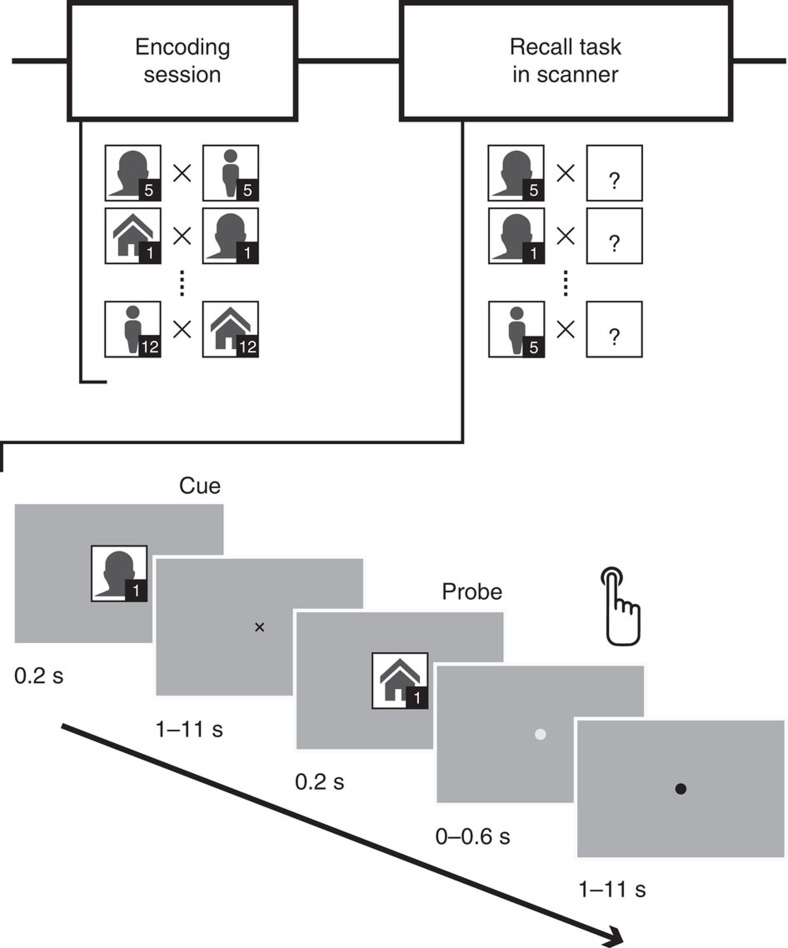
Experimental procedure and trial structure. Top: participants learned the associations between grayscale pictures depicting either a face plus body, scene plus face or body plus scene, during an initial encoding session. Subsequently, participants retrieved these associations in the scanner. Note that in the actual experiment, category icons and pair numbers (used here for illustration purposes) were replaced by photographic stimuli, as described in the Methods. Bottom: each retrieval trial comprised a cue, a retrieval phase of variable length and the presentation of a match or non-match probe stimulus (bottom). Participants indicated whether the probe matched the paired-associate by button press. Trials were separated by a variable inter-trial interval.

Participants performed a paired-associate retrieval task in the MRI scanner after having learned the associations between pairs of grayscale images of faces, houses and faceless bodies (Fig. 1). All participants (*N*=25) were able to remember the associations with high accuracy (average performance: 84.6% correct responses, s.e.m.=2.0%, average d-prime: 1.61, s.e.m.: 0.07).

### Conjunctive representations during memory retrieval

**Figure 2:**
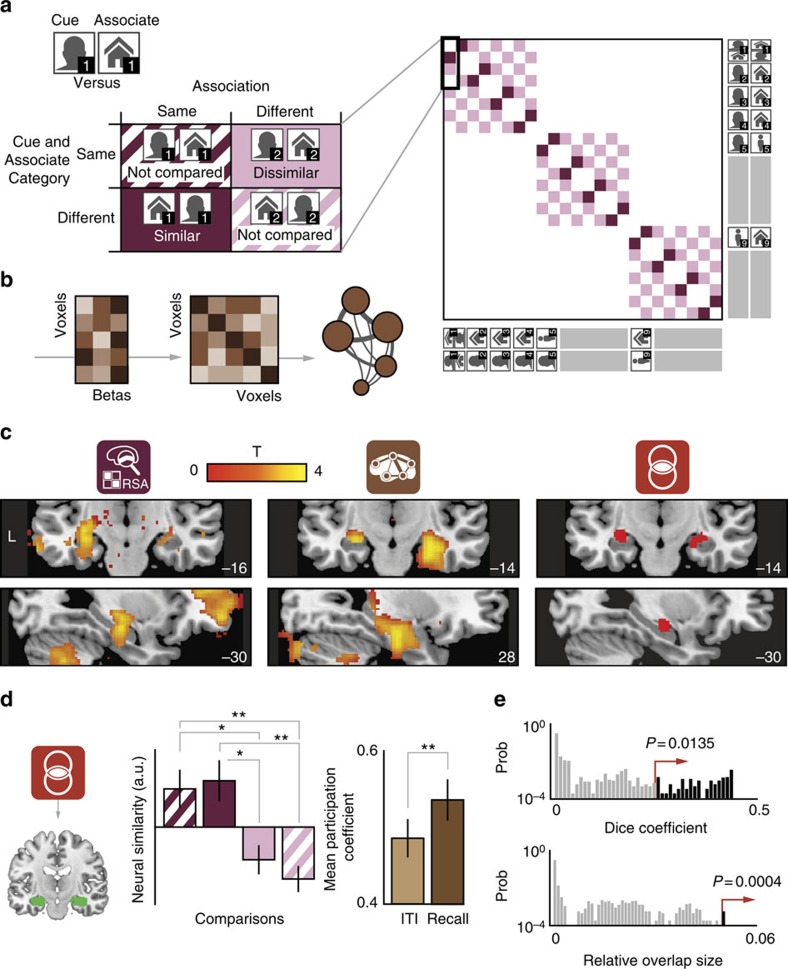
Conjunctiveness and hubness in the hippocampus. (**a**) Representational similarity analysis (RSA) logic. Left: associative similarity contrast, with expected high regional representational similarity for comparisons of the same association, and low similarity for comparisons of different associations, yielding a conjunctiveness metric for each voxel. Specific comparisons were excluded to penalize perceptually driven effects (striped/blank cells): within-association comparisons with identical cue or associate stimulus categories (top left quadrant in matrix), and between-association comparisons with different cue and associate stimulus categories (bottom right quadrant). Right: full condition-by-condition RSA contrast matrix used in the whole-brain searchlight approach. Each cell represents a specific comparison between two conditions. Darkness indicates degree of expected pattern similarity. Four example comparisons are outlined. (**b**) Logic of network analysis. Whole-brain beta time-series correlation (left, five example voxel time series) was performed to obtain a voxel-by-voxel functional connectivity matrix (middle, darker shades indicate higher correlation coefficients). The participation coefficient was computed to obtain a hubness metric for each voxel, reflected by node size in the example graph (right, thickness of the edge relates to connectivity strength). (**c**) Both RSA and network analysis show significant effects in the hippocampus (*P*<0.05 small-volume-corrected, thresholded at *P*<0.05 uncorrected for display purposes) and overlap of both effects. (**d**) Hippocampal voxels showing overlapping effects were selected (left) to extract normalized similarity estimates (middle) for each comparison shown in **a** and hubness scores for ITI and recall periods (right). *P<0.05, **P<0.005. Note that comparisons between these bars are shown for display purposes only and reflect the effect shown in **c** for the selected hippocampal overlap voxels. (**e**) Observed Dice coefficient and relative overlap size (proportion of voxels showing overlap) of the hippocampus and associated *P*-value based on the null-distribution from the label shuffling (spatial resampling) procedure. Histogram *y* axis depicts the probability of observing a certain overlap statistic in randomly selected ROI (prob) on a logarithmic scale. The hippocampus shows significantly more overlap of conjunctiveness and hubness metrics than other regions in the brain.

To test whether the human hippocampus fulfills the criteria of a convergence zone, we specifically aimed to detect overlap of conjunctiveness and hubness. We operationalized conjunctiveness as the amount of information about specific memory associations in patterns of fMRI activity. To this end, we alternated temporal order of cue and paired-associate instances already during learning, to have participants create one conjunctive representation for each pair of stimuli, independent of their order. We then systematically assessed the presence of these conjunctive representations in spherical regions surrounding a single voxel (search lights), using RSA. Specifically, we applied a representational similarity contrast where we expected higher neural pattern similarity when comparing instances of the same association relative to comparing different associations (that is, associative similarity, Fig. 2a). In addition, we imposed a perception penalty on this contrast by excluding perceptually similar comparisons and thereby emphasizing perceptually dissimilar comparisons. As a result, category-related perceptual contributions to pattern similarity were penalized, maximizing the sensitivity of our analysis to detect conjunctive mnemonic representations (see Methods and Fig. 2a for details). The final contrast resulted in a conjunctiveness score for each individual voxel (Fig. 2c left panel). As predicted, the hippocampus showed a significant conjunctiveness effect (peak Montreal Neurological Institute (MNI) coordinates: *x*,*y*,*z*=[−30,−16,−14], *t*_24_=3.59, *P*=0.045 small-volume family-wise error (FWE) corrected using Threshold-Free Cluster Enhancement, see Methods for details). We found no significant differences in other brain regions in a follow-up analysis (*P*>0.17 whole-brain FWE-corrected, see Supplementary Fig. 1A).

### Network centrality during memory retrieval

In parallel, we employed a whole-brain graph theoretical analysis to probe neural network dynamics during associative memory retrieval. To this end, we computed beta time-series correlations between all grey matter voxels in the brain^32^ and summarized the connectivity profile of each voxel into a hubness score^33^ (Fig. 2b, see Methods for details). We used the participation coefficient as our hubness metric, which quantifies the importance of a given node (that is, voxel) for interactions between subnetworks^33^,^34^. Nodes participating in multiple subnetworks (so-called connector hubs) are likely integrating different types of information across distributed brain regions and function as convergence zones^33^. In addition, the participation coefficient provides a more sophisticated and robust index of hubness than traditional measures, such as degree centrality^35^. To obtain a task-related measure of hubness for each voxel, participation coefficients during memory retrieval were contrasted with rest intervals (Fig. 2c middle panel), in the absence of head displacement differences between task phases (see Supplementary Fig. 2 and Methods for details). In line with our predictions, the hippocampus showed a significant retrieval-related hubness effect (peak MNI coordinates: *x*,*y*,*z*=[28,−14,−22], *t*_24_=3.75, *P*=0.009, small-volume FWE-corrected, see Table 1 for a list of other conjunctiveness cluster peaks and their hubness scores for comparison). We found no significant hubness differences in other brain regions in a follow-up analysis (*P*>0.42 whole-brain FWE-corrected, Supplementary Fig. 1B, see Supplementary Fig. 3 for the participation coefficient map from the rest interval only). In addition, to corroborate the participation coefficient results, we repeated the analysis with a different hubness metric, eigenvector centrality, on alternatively preprocessed data (see Methods for details). We observed a similar hippocampal effect (peak MNI coordinates: *x*,*y*,*z*=[32,−18,−16], *t*_24_=3.56, *P*=0.046, small-volume FWE-corrected, Supplementary Fig. 4).

### Overlap between convergence metrics

Finally, to assess the overlap of the hubness and conjunctiveness metrics, we thresholded and binarized both the conjunctiveness and hubness maps, and calculated their intersection. As predicted, we observed overlapping patches of conjunctiveness and hubness in the hippocampus (Fig. 2c right panel, Supplementary Fig. 1C). Next, we defined the set of hippocampal voxels showing overlap on the group level as a region-of-interest (ROI) for *post hoc* analyses (Fig. 2d). As expected, voxels from the overlap ROI showed effects for both conjunctiveness and hubness metrics (see Supplementary Fig. 5 for an exploratory whole-brain connectivity analysis with the overlap ROI as seed region). Moreover, we observed no associative similarity effect for the temporal order in which an association was recalled (*P*>0.26), but recall of the same association was always more similar than recall of a different association, suggesting that the measured conjunctive representations are independent of the temporal order of the stimulus pairs. In addition, we found no evidence for dependence of the associative similarity effect on the type of probe stimulus (*P*>0.25, Supplementary Fig. 6, see Methods for more details). To further investigate the relationship between conjunctiveness and hubness metrics in the overlap ROI, we performed an across-voxel correlation analysis within each participant (see Methods for details). On the group-level average, voxels from the overlap ROI showed significant above-zero correlation coefficients (Wilcoxon signed-rank test: *Z*=2.21, *P*=0.026), indicating a relationship between conjunctiveness and hubness metrics. But, how surprised should one be to observe overlap specifically in hippocampus? To answer this question, we performed a ROI-based spatial resampling procedure, designed to assess whether the observed overlap was greater than potential spurious overlap at a certain threshold and quantify this expression in a *P*-value (see Methods for details). In this analysis, we calculated two complementary overlap statistics, namely Dice overlap coefficient and relative overlap size, using all voxels from the left and right hippocampus (Fig. 2c)^36^, as defined by the Automated Anatomical Labeling atlas for SPM8 (ref. 37). We then compared the resulting overlap metrics to a null-distribution obtained by resampling with randomly permuted region labels using all 116 atlas regions. In other words, for each permutation, we computed the overlap scores for voxels from two randomly selected regions, which yielded our null-distribution. The hippocampus showed significantly more overlap of representation and connectivity metrics (Dice coefficient: .25, *P*=0.0135; relative overlap size: 5%, *P*=0.0004) than expected by chance (Fig. 2e). These results were robust to various cutoff values used to threshold the two maps (Supplementary Fig. 7). Notably, we did not observe this effect when we substituted either the conjunctiveness or hubness map with a univariate activity map where we contrasted the retrieval phase with the inter-trial intervals (univariate with conjunctiveness or hubness: for all combinations Dice coefficient: 0, *P*=1; relative overlap size: 0%, *P*=1, see Supplementary Fig. 8 for whole-brain univariate results). These results suggest that the significant overlap of conjunctiveness and hubness in the hippocampus are not explained by univariate signal differences.

## Discussion

Using a combined approach of representational similarity and network analyses, we provide evidence for mnemonic convergence in the human hippocampus. Our findings highlight the key role of the hippocampus in representing conjunctive information and relate this function to its importance in connecting subnetworks during memory retrieval. We demonstrate that this crucial role of the hippocampus as a connector hub is notably prevalent during memory retrieval, at the same time when conjunctive representations are reactivated^38^.

The present results are in line with both theoretical work and empirical findings. Hippocampal place cells integrate the spatial features characterizing a specific location and have been put forward as the essential elements of a map-like representation of the environment^6^. In addition, other types of high-level conjunctive cells have been observed in the hippocampal formation, such as cells coding for conditioned behavioural responses^39^, specific olfactory cues^40^, the conjunction of location and heading direction of an animal^41^, or location in conjunction with a remembered object^12^. These conjunctive representations constitute the hallmark of episodic memory, as they represent the relations between elements of an episode^7^,^13^. Our results are consistent with the idea that the hippocampus contains these index-like representations^8^ in sparse networks^42^,^43^, binding multiple cortical elements of an episode into memory^10^,^19^,^35^,^44^,^45^,^46^.

Evidence from animal electrophysiology as well as human lesion and anatomical connectivity studies posits that the hippocampus acts as a major network hub during retrieval: the hippocampus ultimately receives input from most regions of the brain via the entorhinal cortex and thus is an anatomical hub^47^,^48^. The hippocampus constitutes the apex of the visual processing hierarchy since it receives converging inputs from most upstream visual regions^21^. Graph-theoretical analyses of human diffusion tensor-imaging data have revealed that the hippocampus is part of a so-called rich-club of network hubs, characterized by denser connectivity among club members than with less connected regions^49^,^50^. This finding is well in line with the results of our network analysis, where we identify the hippocampus as a connector hub during memory retrieval. By using the participation coefficient to quantify hubness from whole-brain connectivity data in our graph-theoretical analysis, we summarize the importance of the hippocampus for interactions between distributed subnetworks. Moreover, by contrasting this hubness metric during retrieval against the inter-trial intervals, we are able to isolate task-related contributions. Note, however, that we used the inter-trial interval as baseline, and therefore the observed relative participation coefficient increase might be due to a hubness decrease during the inter-trial interval. Nevertheless, the observed increase in participation coefficient suggests a more prominent role for the hippocampus during the retrieval of memories, likely represented in distributed parts of the brain. We showed that the hub status of the hippocampus is linked to the memory retrieval phase in our task, which accords with the large body of human neuroimaging evidence implicating the hippocampus in memory retrieval^51^,^52^, as well as recent electrophysiological studies suggesting that the hippocampus serves as a network communication hub for memory^24^,^53^.

Moreover, we provide experimental evidence that these network characteristics of the hippocampus directly relate to its representational role: conjunctive coding of associative information. We observe both hub-like properties and conjunctive representations in the hippocampus, suggesting that the hippocampus acts as a convergence zone. This notion fits with computational models and general principles of brain function^54^, which recognize convergence as a key motif in the brain^55^. Information about the external world is processed by sensory regions and progressively integrated as it reaches upstream brain areas and is ultimately evaluated by decision-making systems^56^. By demonstrating the hub role of the hippocampus during memory retrieval, we provide a strong link between functional connectivity and functional specialization for memory processes: as an important connector hub, the hippocampus is able to integrate information from multiple subnetworks into a coherent conjunctive representation, consistent with the convergence motif.

Although we did not aim to investigate functional specialization along the hippocampal long-axis, we observe overlap between representation and connectivity in the middle and anterior part of the hippocampus. There is substantial evidence for a functional specialization along the posterior–anterior axis of the hippocampus^57^,^58^,^59^. Our findings may relate to the preference of the anterior hippocampus for non-spatial stimulus material or more abstract, higher-level associative information^59^, such as temporal order-invariant conjunctions relevant in the current experiment^60^.

In conclusion, we show that the human hippocampus acts as a mnemonic convergence zone, characterized by both hub-like network connectivity and conjunctive representations. We thereby provide evidence for the long-held hypothesis that the hippocampus binds distributed information into memories. Furthermore, we outline a quantitative method to investigate convergence zones in humans, whose existence has been hypothesized for a long time by computational models. Future applications of our approach could leverage this method to track the dynamics of hippocampal processing during memory consolidation and to investigate the integrity of the hippocampus during normal or pathological ageing.

## Methods

### Participants

Thirty-five participants (19 females, average age: 22.7 years, range: 18–32 years) took part in the study. All were in good health, with no history of psychiatric or neurological diseases, no brain abnormalities and normal or corrected-to-normal vision. Before the experiment, participants gave their informed consent and were reimbursed for their participation. All experimental procedures were approved by the local ethical review committee (CMO region Arnhem-Nijmegen, The Netherlands). Five participants were excluded due to technical problems with the scanner and an additional five participants since they were unable to reach a sufficient performance level (d-prime<1.0). Therefore, the data of 25 participants (15 females, average age: 22.7 years, range: 18–32 years) entered our analysis. The sample size was based on previous RSA studies on memory and the hippocampus^11^,^59^.

### Stimuli

We used grayscale images of faces (Karolinska Directed Emotional Faces)^61^, houses (Stanford Vision Lab stimulus set)^62^ and human bodies (Bodily Expressive Action Stimulus Test set)^63^. All images were cropped to 200 × 200 pixel dimensions and normalized using the SHINE toolbox for MATLAB^64^ (v2014a, The MathWorks) by adjusting the mean luminance and s.d. of the intensity values for each pixel. Stimuli were presented to participants using the Presentation software package (v16.4, Neurobehavioral Systems).

### Paired-associate learning before the scanning experiment

Participants commenced with the initial encoding session outside the scanner, separated into six study and test cycles. During study cycles, participants learned 12 random associations between pairs of pictures. Associations comprised face–house, face–body and house–body pairs (four pairs of each type). In a study block, the 12 pairs were presented in random order. In each trial, the two stimuli of each pair were shown in succession (1,000 ms on-screen, 1,000 ms inter-stimulus interval). We used an inter-trial interval of 3,000 ms, during which a fixation dot was presented. Order of presentation of the two stimuli per pair was counterbalanced across cycles.

In the test blocks, 48 test trials were presented in which one of the stimuli of each pair was presented as a retrieval cue, followed by a probe stimulus, which could either be the associate (match probe) or a different stimulus from the same category (non-match probe). Either of the pair members could appear as a cue, with the order counterbalanced within and across cycles. The cue and probe stimuli were each presented for 200 ms. Cue and probe presentations were separated by a retrieval phase of 1,000, 3,000 or 5,000 ms (counterbalanced across cues, pairs, matching probe and cycles) during which participants were asked to retrieve the specific associate of the cue. Participants were instructed to respond as fast as possible with their right hand, using two response buttons, and to indicate whether the probe matched the associate (hit or false alarm) or not (correct rejection or miss). Response mapping of these two buttons was counterbalanced across participants. The maximal response window was set to 600 ms. If participants did not respond within the response window, then a too-late message was presented for 1,000 ms. The variable retrieval phase together with the short response window ensured that participants had to respond promptly to elicit immediate memory retrieval. After each response, feedback was provided by presenting the associate (1,000 ms on screen). Trials were separated by variable inter-trial intervals of 1,000, 3,000 or 5,000 ms (retrieval phase and inter-trial interval added up to 6,000 ms in each trial). During a given test block, each association was tested 4 times. At the end of each test block, the percentage of correctly responses was displayed to the participant. We encouraged participants, by way of a monetary reward (a bonus of 5 Euros), to reach a minimum of 80% correct responses (hits and correct rejections) in at least one of the test blocks, in order to foster high memory performance.

### Retrieval task in the scanner

After a 30-min break, participants performed the retrieval task in the MRI scanner in 2 runs of ∼25 min each, with a short half-time break in-between lasting ∼5 min. During the scan session, a total of 288 retrieval test trials were presented to the participant (144 trials in each run). Trial structure was identical to the combined test blocks of the encoding session. However, we did not provide feedback and set the retrieval phase and inter-trial interval lengths to 1,000, 6,000 and 11,000 ms, respectively. The performance score was only displayed at the end of the experiment. The pairs were presented 12 times in each run: 6 times for each of the two possible temporal cue-associate orders. Conditions, trial durations and match probes were counterbalanced within each run. Trial order was randomized in both runs.

### Data acquisition

Neuroimaging data were acquired using a 3-T MR scanner (TIM Trio; Siemens Healthcare) in combination with a 32-channel head coil. For the functional scans, we used a three-dmensional (3D) echo planar imaging (EPI) sequence (voxel size: 2 × 2 × 2 mm, volume TR: 1,800 ms, TE: 25 ms, flip angle: 15 degrees, 64 slices, FOV: 224 × 224, orientation: −25 degrees from transverse plane, GRAPPA acceleration factor: 2, acceleration factor 3D: 2)^65^. Using the AutoAlign head software by Siemens, we ensured a similar FOV tilt across participants. Functional scan runs contained between 1032 and 1093 volumes, since the instruction screens were self-paced. In addition, we acquired field maps using a gradient echo sequence (voxel-size: 3.5 × 3.5 × 2 mm, volume TR: 1020, ms, TE1: 10.00 ms, TE2: 12.46 ms, flip angle: 90 degrees, 64 slices, FOV: 224 × 224, orientation adjusted to functional sequence, descending slice order). At the end of the scanning session, we obtained a structural scan using an MPRAGE sequence (voxel-size: 1 × 1 × 1 mm, volume TR: 2,300 ms, TE: 3.03 ms, flip angle: 8 degrees, FOV: 256 × 256, ascending slice order, GRAPPA acceleration factor: 2, duration: 5:21 min).

### fMRI preprocessing

We preprocessed MRI data using the Automatic Analysis framework (https://github.com/rhodricusack/automaticanalysis/wiki), which combines tools from SMP8 (http://www.fil.ion.ucl.ac.uk/spm/software/spm8/), FreeSurfer (v5.1, http://surfer.nmr.mgh.harvard.edu/) and the FMRIB Software Library (v5.0, http://fsl.fmrib.ox.ac.uk/fsl/fslwiki/), complemented by custom scripts. The preprocessing pipeline consisted of the following steps: we removed biases resulting from field inhomogeneities from the native structural images using the SPM8 new segment option. Furthermore, we denoised the structural images using an Adaptive Optimized Nonlocal Means filter (MRI denoising software package)^66^. Next, we performed a premasking procedure to exclude the neck from the structural image using a template image and ran a Freesurfer brain extraction and SPM segmentation procedure to obtain segmentation masks for grey matter, white matter, cerebrospinal fluid and out-of-brain voxels. Furthermore, we realigned and unwarped the functional images using the fieldmap images. In addition, we employed a spike-detection algorithm to record and later model signal spike events as nuisance variables. Functional and structural images were coregistered to a functional template (mean EPI) and a structural template respectively, after which the functional images were registered to structural space. We extracted the signal time course from white matter, cerebrospinal fluid and out-of-brain voxels and included these as nuisance variables. Field bias was removed from the mean EPI after which we performed a Freesurfer brain extraction procedure to obtain a brain mask. To account for inter-subject differences in brain morphology, we constructed a group structural template using the Advanced Normalization Tools toolbox (v1.9, http://stnava.github.io/ANTs/). Subsequently, we used the parameters obtained via this procedure to later normalize our single-subject statistical maps to an intermediate common space as a final step, before transforming to MNI space using FMRIB's Linear Image Registration Tool and performing the group-level statistical analysis.

### General linear modelling

Our main analyses were restricted to the retrieval phase in each trial and we included all 288 trials in our analyses. We used all trials (trials with correct responses and the small number of trials with incorrect responses) since we aimed to obtain the most reliable estimate of response patterns, by constructing balanced regressors containing three trials of each condition: we modelled brain activity during the retrieval phase and inter-trial intervals by using three randomly selected trials with a short, medium and long duration from the same run and condition (144 trials, resulting in 48 regressors per functional run) using boxcar functions spanning the respective intervals. The three trials selected for a given condition were maximally spaced apart in time. By modelling three trials with different retrieval phase durations from the same condition, with different onset spreads across the experiment, we aimed to minimize influence of time-dependent effects, such as temporal autocorrelation and habituation effects, and thereby obtain a more reliable set of beta estimates for each experimental condition. We included the small number of incorrect response trials to be able to balance the total amount of delay for each regressor (one short, one medium and one long trial) and be able to utilize the short-delay trials, at the expense of making our analysis potentially more conservative. Inter-trial intervals were explicitly modelled to obtain the beta estimates required for the network analysis. For each condition-specific regressor (containing the retrieval phases of three delay intervals), we ran a general linear model (GLM) including the regressor-of-interest and one single additional regressor containing all other conditions and other task (that is, regressors for faces, scenes, bodies, probes, retrieval cues and button presses) and nuisance variables^67^, using standard SPM functions with default settings. Both runs were modelled together in each GLM, accounting for general differences between the runs. In total, we obtained the beta images for 96 retrieval phase regressors and another 96 complementary inter-trial interval regressors (48 per functional run). Decorrelating regressors for different groups of trials from the same condition using this iterative method yields beta weights well-suited for multivariate pattern analysis on event-related designs^67^.

### Searchlight representational similarity analysis

We performed a whole-brain searchlight analysis to assess which regions contained multivoxel information about specific memory representations^30^. After applying a grey matter mask, we extracted the multivoxel activity pattern within each spherical searchlight (4 voxel radius, including a minimum of 30 grey matter voxels), from each of the 96 retrieval phase beta images. Similarity between patterns was computed using Spearman's correlation to account for nonlinear effects and deal with outliers without specifying an arbitrary threshold^68^. We then constructed a balanced regressor-by-regressor contrast matrix for the hypothesized representational similarity pattern, with a mean value of 0. The observed similarity space of each sphere was then fitted to the contrast matrix, using a GLM. The resulting parameter estimates were assigned to the centre voxels of each sphere. To correct potential biases in the *T*-value distributions and to equalize variance across participants, we applied a mixture model to our *T*-maps. We then warped the resulting statistical maps to MNI space and performed additional smoothing (full-width at half maximum (FWHM): 2 mm) to improve spatial alignment across participants.

### Conjunctive mnemonic information contrast

To be sensitive to conjunctive memory retrieval in our analysis, we defined a specific contrast where we expected high pattern similarity when comparing the multivoxel activity patterns of a specific association to a different instance of the same association (associative similarity contrast, Fig. 2a). Conversely, when we compared the patterns during retrieval of a specific association with the pattern in response to a different association, we expected high dissimilarity. To control for unspecific perceptual effects and to maximize our sensitivity for mnemonic representations, we introduced a perception penalty by excluding specific comparisons: whenever we compared neural patterns of two instances of the same association, the cue-associate order of one of the instances was always reversed. Conversely, when we compared instances of different associations, we made sure that cue-associate order was identical. Any perceptual similarity effects driven by the visual categories of the cue and associate were thus minimized.

### Functional connectivity analysis

For the connectivity analysis, we concatenated beta estimates for regressors of the retrieval phases (used for the RSA) and inter-trial interval separately, resulting in two beta vectors per voxel. After spatial subsampling (resulting in a voxel size of 8 × 8 × 8 mm) we computed voxel-wise spatial correlation coefficients of the beta vectors to quantify functional connectivity for each condition. All following analyses were performed on the weighted connectivity matrices, where negative correlations were set to zero^33^ and all positive edges were thresholded at *P*<0.05 (false discovery rate corrected), to preserve significant connections. We indexed hubness by estimating the participation coefficient, quantifying the distribution of voxel-wise connections among local subnetworks. To assign each voxel to a subnetwork, we derived an additional 116 × 116 region-by-region connectivity matrix from the averaged beta vectors, where regions were defined using the Automated Anatomical Labeling (AAL) atlas^37^. Subsequently, after thresholding (edges>0, *P*<0.05 false discovery rate-corrected) we parcellated the 116-node network using modularity detection (Louvain method^69^) and assigned each voxel to one of the resulting subnetworks. We computed the participation coefficient PC for each voxel *i* by closely following the procedure employed by Power and colleagues^33^. PC is given by:


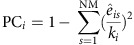


Here *ê*_*is*_ is the number of edges of voxel *i* to voxels in subnetwork *s*, while *k*_*i*_ is the total amount of connections of voxel *i*, and *NM* is the number of subnetworks. This procedure resulted in a normalized voxel-wise measure ranging from 0 (provincial hub: only connecting within subnetwork) to 1 (connector hub: only connecting between subnetworks). Next, we transformed the hubness maps to MNI space and contrasted the retrieval phase with the inter-trial intervals (Supplementary Fig. 1B).

### Statistical analysis of conjunctiveness and hubness maps

To test whether voxels in the hippocampus show significant effects, we used FSL RANDOMISE to obtain nonparametric statistics with 10,000 random permutations. The test statistic was based on a one-sided *t*-test of within-subject difference maps, with 5-mm variance smoothing and threshold-free cluster enhancement^70^. We corrected for multiple comparisons using FWE correction, restricted to a small-volume comprising bilateral hippocampus, as defined by the AAL atlas. All whole-brain maps presented in the current work were thresholded with voxel-wise nonparametric *P*-values obtained using FSL RANDOMISE. We obtained *post hoc* modelled mean pattern similarity estimates for the separate comparisons (for example, same association with same order, same association with different order, different association with same order, and different association with different order) using four contrasts of the isolated comparisons against zero. We fitted these contrasts using a GLM and averaged the beta estimates, reflecting neural similarity, from all hippocampal voxels showing overlap of the RSA and functional connectivity analysis (in volume space, see the overlap ROI, Fig. 2d). The magnitude of these beta estimates was then normalized by demeaning across the four conditions within each participant. In addition, we extracted the hubness estimates from the overlap ROI for the ITI and recall periods. Comparisons between these measures (*P*-values obtained using two-tailed nonparametric paired *t*-tests with 100.000 permutations) were added for display purposes (Fig. 2d). To investigate the relationship between hubness and conjunctiveness metrics, we computed Spearman's correlation coefficients across voxels from the overlap ROI. Spearman's coefficients were used to account for nonlinear effects. Next, we tested for a significant positive or negative relationship on the group level, using a two-tailed Wilcoxon signed-rank test. For visualization of the imaging results, whole-brain cortical and cerebellar surfaces renderings were created using the brain visualization tool CARET (v5.65, http://brainmap.wustl.edu/caret.html). Note that these surface renderings were only used for visualization. All statistical tests were performed on the volume maps. To illustrate the main effect in the hippocampus, volume maps are shown in Fig. 2c.

### Statistical analysis of overlap between convergence metrics

To test regional coincidence of hubness and conjunctiveness, we opted for a hypothesis-driven, yet full-brain resampling approach: first, we defined our predicted bilateral hippocampal ROI as the corresponding anatomical masks extracted from the AAL atlas. Next, we computed summary overlap statistics for our anatomical hippocampal ROI. We binarized our voxel-wise network centrality map in MNI space, yielding a binary vector **H** defining so-called hub voxels for our anatomical ROI. This procedure was repeated for the conjunctive information map to obtain a binary vector **C** defining the informative voxels in the hippocampus. Both the conjunctiveness and hubness maps were thresholded at *P*<0.05 uncorrected, using the voxel-wise nonparametric *P*-values. We computed two complementary metrics to quantify overlap: first, we used the Dice coefficient to assess the specificity of overlap between hubness and conjunctiveness effects, regardless of extent and region size. Here double the length of the logical conjunction between **H** and **C** is divided by their summed individual lengths (that is, the sum of all logical true elements in both vectors separately):


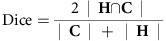


Second, to quantify the extent of overlap, relative to the total region size, we computed proportion of voxels that show both hubness and conjunctiveness effects of our hippocampal ROI containing a total number of voxels *n*, using the following equation:


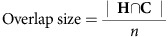


This procedure yielded two complementary overlap measures for the left and right hippocampus, on which we subsequently performed a spatial permutation test. Here we computed the same overlap score for randomly selected ROIs with 10,000 permutations (two random subregions from the AAL atlas in each permutation). For the crucial final statistical test, we hypothesized that no >5% of all of these random ROIs would yield overlap scores higher than the overlap scores observed in bilateral hippocampus. We investigated whether the cutoff nonparametric *P*-value used to threshold the input vectors *H* and *C* influenced the results. To this end, we repeated the procedure and plotted the corresponding probability of observing a higher overlap score in a random ROI as a function of the critical *P*-value used to threshold the input vectors (Supplementary Fig. 7).

### Univariate activity contrast

To test whether effects resulting from hubness or conjunctiveness metrics could be explained by univariate effects, we smoothed our data (FWHM: 8 mm) and applied a GLM including regressors for retrieval phases, inter-trial intervals, faces, scenes, bodies, probes, retrieval cues and button presses for each functional run. Next, we contrasted the beta images of the retrieval phases with the beta images of the inter-trial intervals (Supplementary Fig. 8). Univariate activity maps were analysed in the same way as the conjunctiveness and hubness maps, that is, warped to MNI space via Advanced Normalization Tools common space, before obtaining nonparametric statistics.

### Head displacement analysis

To rule out potential head movement biases in our network analysis^26^, we compared the root-mean-square of all six translation parameters of the retrieval and inter-trial intervals. A *t*-test revealed no significant differences between conditions (*t*_24_=0.05, *P*=0.96). In addition, a histogram of mean displacements magnitudes revealed no apparent differences on a finer scale (Supplementary Fig. 2).

### Eigenvector centrality analysis

To corroborate our participation coefficient results and evaluate the robustness of our connectivity findings, we repeated our analysis with a different centrality measure and alternative preprocessing. Here we followed the procedures used by Ekman *et al*.^26^: we extracted coregistered time series from all grey matter voxels and shifted the time course by 3 volumes (5.4 s) to compensate for the hemodynamic response lag. We regressed out head motion and out-of-brain signal from the time series, followed by a spatial subsampling procedure, resulting in a voxel size of 4 × 4 × 4 mm. Next, we computed voxel-wise spatial correlation coefficients of the retrieval phases and inter-trial intervals separately. All subsequent analyses were performed on the weighted connectivity matrix, where negative correlations were set to zero. We derived a centrality score for each individual voxel by computing the eigenvector of the connectivity matrix with the highest eigenvalue. Compared with the participation coefficient, eigenvector centrality is a coarser hub measure, that indicates how important (that is, central) regions are within the global network. We followed a procedure similar to the participation coefficient analysis, where we transformed the eigenvector centrality maps to MNI space and contrasted retrieval phase with the inter-trial intervals (Supplementary Fig. 3).

### Seed-based connectivity analysis

For the exploratory seed-based connectivity analysis, we used the same recall and ITI beta time-series constructed for the network analysis. We back-warped the ROI mask with the hippocampal overlap voxels (Fig. 2d) to individual participant brain space. After applying spatial smoothing (FWHM: 8 mm), we extracted the mean time course of the overlap ROI and computed spatial correlation coefficients with all brain voxels. Coefficients of the recall and ITI phases were warped to MNI space, Fisher's *Z* transformed and contrasted, to obtain a normalized whole-brain difference map (Supplementary Fig. 5).

### Probe type control analysis

Although we excluded the probe presentation interval from our recall regressors and explicitly modelled probe stimuli as nuisance in our initial GLM, it is important to investigate the influence of probe type: when comparing two matching-probe trials of the same association, but with different order, participants ultimately view the same two stimuli, whereas in the non-match probe trials only the cue stimulus is shared. Therefore, as we argue that our RSA is sensitive to mnemonic representations, the associational similarity effect should not be predominantly driven by the match probe trials. To assess whether our associative similarity effect in the hippocampal overlap ROI is driven by probe type, we performed an additional GLM analysis with separate regressors for match and non-match probe trials (Supplementary Fig. 6). The obtained similarity estimates for the two main comparisons of interest (that is, same association with different order, different association with same order, see Fig. 2a) were demeaned and contrasted (*P*-values obtained using two-tailed nonparametric paired *t*-tests with 100,000 permutations). Note that these match and non-match contrasts are less sensitive, since they are based on half the amount of comparisons entering the main associative similarity contrast.

### Data availability

Data are available from the corresponding author upon request.

## Additional information

**How to cite this article:** Backus, A.R. *et al*. Mnemonic convergence in the human hippocampus. *Nat. Commun.* 7:11991 doi: 10.1038/ncomms11991 (2016).

## Supplementary Material

Supplementary Information Supplementary Figures 1 - 8 and Supplementary References

## Figures and Tables

**Table 1:** List of brain regions representing conjunctive information and their hubness scores.

		Peak MNI coordinates (mm)			Peak *t*-value	
		*X*	*Y*	*Z*	C	H
Anatomical region						
Right	Supramarginal	64	−40	40	4.63	−0.05
Right	Frontal inf tri	54	28	30	4.38	0.57
Left	Angular	−44	−58	32	4.37	−0.28
Left	Precuneus	−8	−62	44	3.83	−1.01
Left	Temporal mid	−52	−40	−8	3.74	1.60
Left	Hippocampus	−32	−16	−8	3.64	0.93
Left	Supp motor area	−12	−10	54	3.31	−0.52
Left	Cerebellum 6	−12	−64	−28	2.79	0.82
Right	Thalamus	12	−12	4	2.47	0.73
Right	Hippocampus	30	−16	−14	2.32	2.77
Right	Temporal mid	54	−18	−10	2.25	−0.98
Left	Temporal mid	−58	6	−30	2.22	0.00
Left	Temporal inf	−64	−58	−8	2.08	0.66
Right	Temporal inf	36	4	−48	1.54	0.23
Right	Precuneus	8	−76	60	1.51	1.09
Right	Cerebellum crus1	58	−64	−34	0.48	0.46
Left	Precentral	−40	−24	72	0.35	0.41
						
Hippocampal region-of-interest						
Left	Hippocampus (C-peak)	−30	−16	−14	3.59	3.07
Right	Hippocampus (H-peak)	28	−14	−22	1.71	3.75

Inf, inferior; mid, middle; tri, triangular.

Table denotes clusters with a minimal extent of 30 voxels from the whole-brain conjunctiveness map, sorted on conjunctiveness peak *t*-value and thresholded at *P*<0.05 (nonparametric). Peak values for conjunctiveness (C, see Supplementary Fig. 1A) and hubness (H, see Supplementary Fig. 1B) are displayed with their coordinates in Montreal Neurological Institute space. Nearest region labels were obtained using the AAL atlas. Statistics of the two hippocampal peak locations reported in the main text (one for conjunctiveness in left hippocampus and one for hubness in right hippocampus, see Fig. 2c) are denoted at the bottom for comparison.
