# Supplementary material for: Mnemonic convergence in the human hippocampus
Source: Nat Commun. 2016 Jun 21;7:11991. doi: 10.1038/ncomms11991 (PMC4919533; doi:10.1038/ncomms11991)
Supplement: Supplementary Information — Supplementary Figures 1 - 8 and Supplementary References [file ncomms11991-s1.pdf]

Supplementary Information

Supplementary Figures

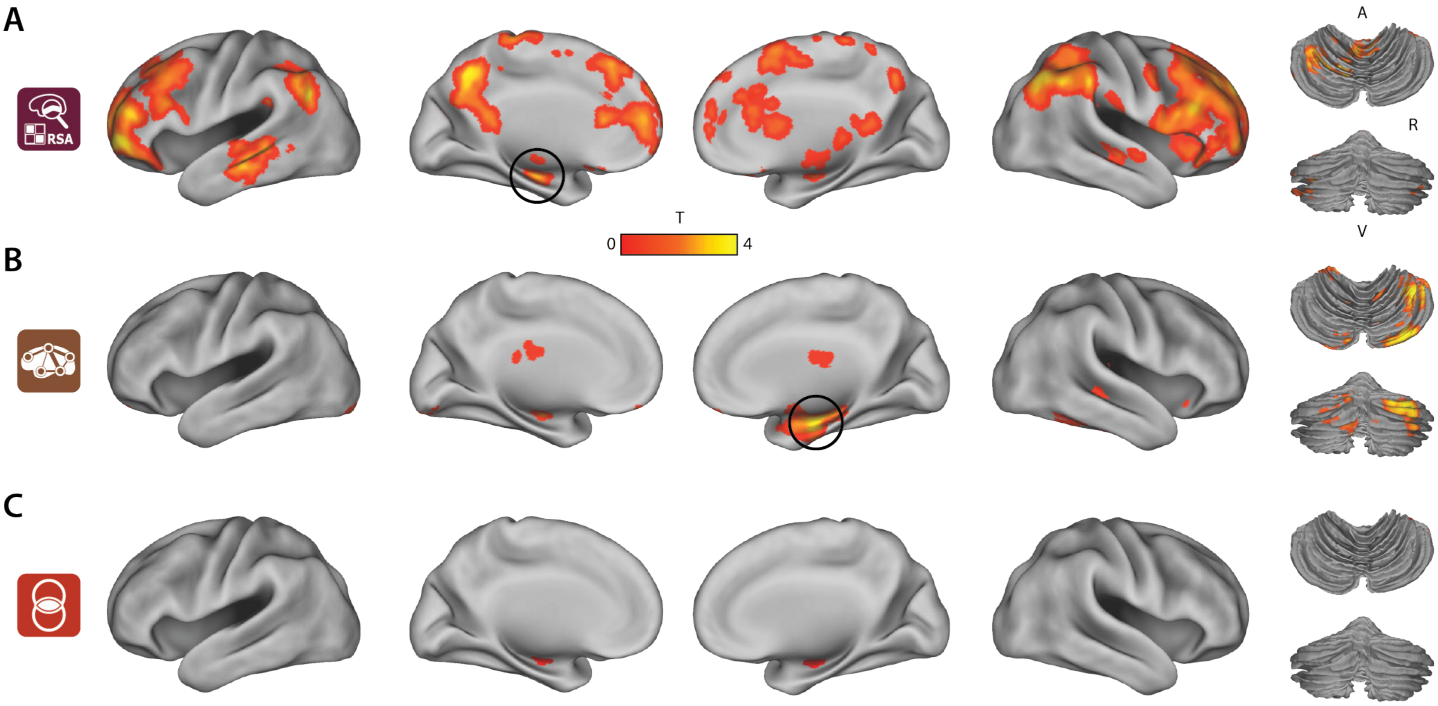

Supplementary Figure 1. Distribution of conjunctive mnemonic information, increased hubness and overlap across the brain. (A) Whole-brain conjunctiveness scores from the RSA, rendered on a cortical surface map. These surface renderings were only used for visualization; the statistical tests were carried out on volume maps. Regions with a high T-value exhibit higher neural pattern similarity when comparing instances of the same association relative to comparing different associations (associative similarity, see Fig. 2A). In addition to the hippocampus, evidence for conjunctive coding was observed in an extended network of regions that have been previously implicated in memory processes, including prefrontal cortex [1-8], lateral parietal cortex [9], precuneus [10] and lateral temporal cortex [11,12]. Note the absence of high values in early visual regions, indicating that the RSA is not sensitive to visual category effects. (B) Whole-brain participation coefficient hubness scores. Regions with a high T-statistic are prominent connector hubs during memory retrieval and therefore relatively important for interactions between subnetworks. The reverse contrast (inter-trial interval versus retrieval condition participation coefficients) yielded no significant differences ($p>.79$ whole-brain FWE-corrected) and no clusters with a minimal extent of 30 voxels thresholded at $p<.05$. Maps in (A) and (B) thresholded at $p<.05$ to illustrate which voxels were used to compute the overlap scores for the spatial permutation test (see Fig. 2E). Circles indicate $p<.05$ small-volume FWE-corrected peaks in the hippocampus. (C) Binary overlap between hubness and conjunctiveness maps, obtained by intersecting (A) and (B). Note that overlap is exclusive to bilateral hippocampus.

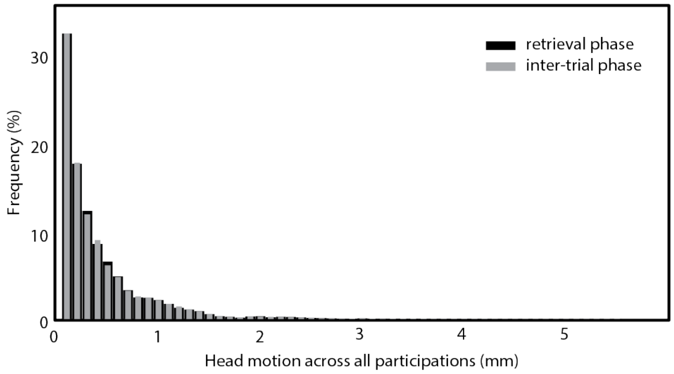

Supplementary Figure 2. Head displacement during retrieval and inter-trial intervals. Histogram of average head movements across participants derived from the realignment parameters. No significant difference was observed between conditions (T_{24}=.05; p=.96, see Methods for details).

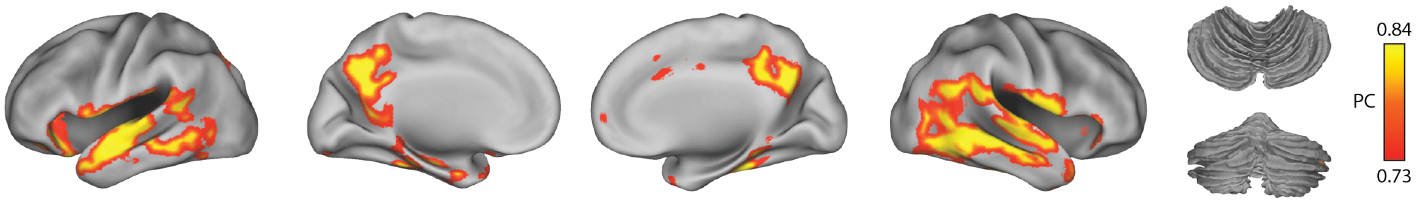

Supplementary Figure 3. Participation coefficient scores during inter-trial intervals. We observed regions previously associated with high participation coefficient (PC) scores during rest blocks, such as lateral temporal gyrus, posterior cingulate, fusiform gyrus, insula and inferior frontal gyrus [13]. Map was thresholded by setting all voxels below 99% of robust range (of non-zero voxels) to zero.

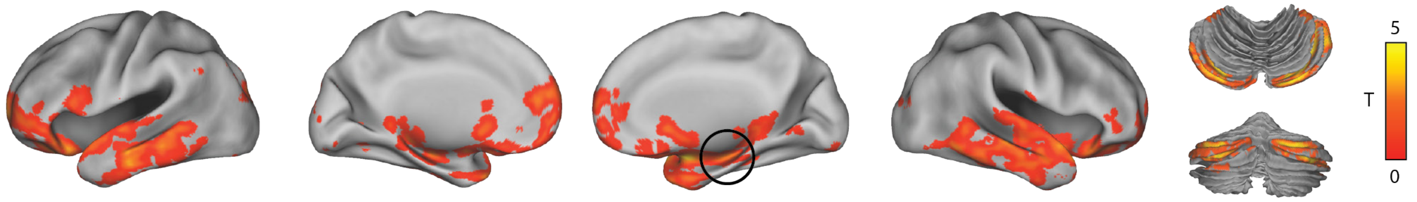

Supplementary Figure 4. Whole-brain eigenvector centrality scores. Recall versus inter-trial interval difference map computed from raw time series data (see Methods for details). Effects are similar to the participation coefficient results (see Supplementary Figure 1). Map thresholded at p<.05 for comparison. Circle indicates p<.05 small-volume FWE-corrected peak in the hippocampus.

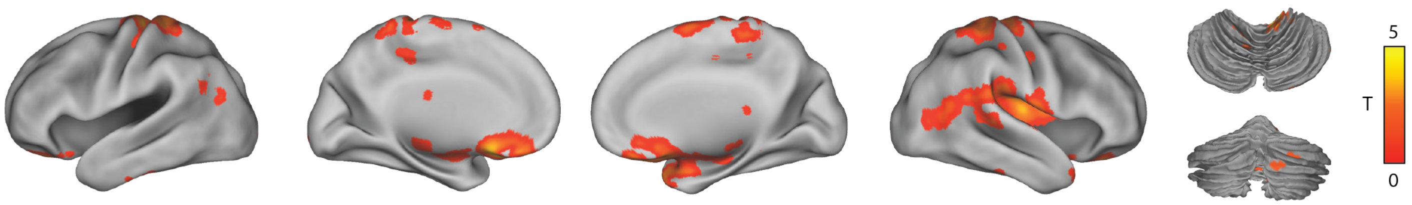

Supplementary Figure 5. Seed-based connectivity profile of hippocampal overlap voxels.
To investigate the functional connectivity of the hippocampus with other brain regions during retrieval, we performed an exploratory seed-based connectivity analysis, with the voxels in the hippocampus that showed effects for both hubness and conjunctiveness (see Fig. 2C, right panel) as seed ROI. Map shows brain regions that potentially drive the observed participation coefficient increase during memory retrieval. We observed no significant differences ($p>.30$ whole-brain FWE-corrected). However, inspection of the seed-based connectivity map at a more liberal threshold ($p<.05$) revealed increases of hippocampal connectivity with an extended network of task-related brain regions, including ventromedial prefrontal cortex [8], sensorimotor cortices and parietal association areas [9,14]. Note that due to this liberal threshold, the results from this analysis should be interpreted with caution.

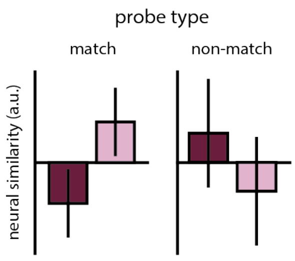

Supplementary Figure 6. Associative similarity effect split for match and non-match probe types. Results from an additional exploratory GLM analysis to assess associative similarity for trials with a probe that matched the associate stimulus separately from trials with a probe that was a different stimulus from the same category. Bars are displaying estimates from the hippocampal overlap voxels (see Fig. 2D). Note that these comparisons (dark purple: association with different order, light purple: different association with same order, see Fig. 2A) are based on half the amount of trials compared to the associative similarity effect presented in the main text. The non-match condition appears consistent with the associative similarity effect, although we observed no significant differences in either of the probe type conditions ($p>.25$). The apparent (but non-significant) reversal of the effect for match trials with respect to the main analysis, in which match and non-match trials are combined, is likely due to the differing variance of the match and the non-match trials.

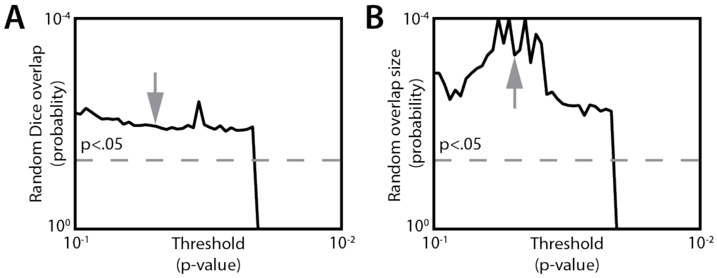

Supplementary Figure 7. Overlap at various thresholds. The impact of different thresholds applied to the conjunctiveness and hubness maps on the specificity of the effect in the hippocampus for the overlap statistics. Logarithmic Y-axis denotes the probability of observing a higher overlap score in random regions-of-interest at a given threshold of the brain maps, plotted on the logarithmic X-axis. Values above the gray dotted line indicate (A) significantly more Dice or (B) relative overlap of conjunctiveness and hubness metrics in hippocampus. Hippocampus generally shows significantly more overlap than random brain regions, unless the applied threshold becomes too conservative (p<.02). Gray arrows indicate the threshold used in the main analysis (p<.05).

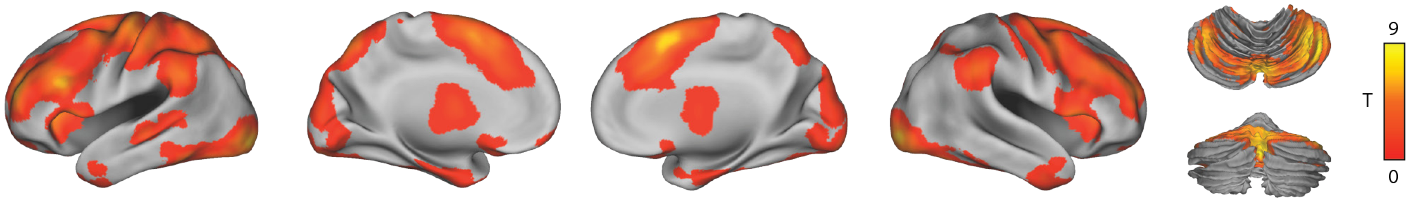

Supplementary Figure 8. Brain regions showing more activation during memory retrieval compared to the inter-trial intervals. Results from a univariate GLM analysis contrasting retrieval versus inter-trial interval activity. Map thresholded at p<.05 to illustrate which voxels contributed to the overlap scores. Note that the hippocampus does not show increased univariate activity during retrieval, even when adopting a liberal threshold.

Supplementary References

1 Ranganath, C., Johnson, M. K. & D'Esposito, M. Left anterior prefrontal activation increases with demands to recall specific perceptual information. *J Neurosci* 20, RC108 (2000).

2 Henson, R. N., Shallice, T. & Dolan, R. J. Right prefrontal cortex and episodic memory retrieval: a functional MRI test of the monitoring hypothesis. *Brain* 122 (Pt 7), 1367-1381 (1999).

3 Nolde, S. F., Johnson, M. K. & Raye, C. L. The role of prefrontal cortex during tests of episodic memory. *Trends Cogn Sci* 2, 399-406 (1998).

4 Rugg, M. D., Fletcher, P. C., Chua, P. M. & Dolan, R. J. The role of the prefrontal cortex in recognition memory and memory for source: an fMRI study. *Neuroimage* 10, 520-529, doi:10.1006/nimg.1999.0488 (1999).

5 Wagner, A. D., Pare-Blagoev, E. J., Clark, J. & Poldrack, R. A. Recovering meaning: left prefrontal cortex guides

controlled semantic retrieval. *Neuron* 31, 329-338 (2001).

6 Buckner, R. L. Beyond HERA: Contributions of specific prefrontal brain areas to long-term memory retrieval. *Psychon Bull Rev* 3, 149-158, doi:10.3758/BF03212413 (1996).

7 Miller, E. K. The prefrontal cortex and cognitive control. *Nat Rev Neurosci* 1, 59-65, doi:10.1038/35036228 (2000).

8 Euston, D. R., Gruber, A. J. & McNaughton, B. L. The role of medial prefrontal cortex in memory and decision making. *Neuron* 76, 1057-1070, doi:10.1016/j.neuron.2012.12.002 (2012).

9 Wagner, A. D., Shannon, B. J., Kahn, I. & Buckner, R. L. Parietal lobe contributions to episodic memory retrieval. *Trends Cogn Sci* 9, 445-453, doi:10.1016/j.tics.2005.07.001 (2005).

10 Fletcher, P. C. *et al.* The mind's eye--precuneus activation in memory-related imagery. *NeuroImage* 2, 195-200 (1995).

11 Binder, J. R. & Desai, R. H. The neurobiology of semantic memory. *Trends Cogn Sci* 15, 527-536, doi:10.1016/j.tics.2011.10.001 (2011).

12 Patterson, K., Nestor, P. J. & Rogers, T. T. Where do you know what you know? The representation of semantic knowledge in the human brain. *Nat Rev Neurosci* 8, 976-987, doi:10.1038/nrn2277 (2007).

13 Power, J. D., Schlaggar, B. L., Lessov-Schlaggar, C. N. & Petersen, S. E. Evidence for hubs in human functional brain networks. *Neuron* 79, 798-813, doi:10.1016/j.neuron.2013.07.035 (2013).

14 van Hoesen, G. W., Pandya, D. N. & Butters, N. Cortical afferents to the entorhinal cortex of the Rhesus monkey. *Science* 175, 1471-1473 (1972).
